# Sex differences in the association of treatment-resistant schizophrenia and serum interleukin-6 levels

**DOI:** 10.1186/s12888-023-04952-0

**Published:** 2023-06-27

**Authors:** Jingqi He, Yisen Wei, Jinguang Li, Ying Tang, Junyu Liu, Zhangyin He, Risheng Zhou, Xingtao He, Honghong Ren, Yanhui Liao, Lin Gu, Ning Yuan, Xiaogang Chen, Jinsong Tang

**Affiliations:** 1 Department of Psychiatry, Sir Run-Run Shaw Hospital, School of Medicine, Zhejiang University, Hangzhou, China; 2 Department of Psychiatry, National Center for Mental Disorders, National Clinical Research Center for Mental Disorders, The Second Xiangya Hospital of Central South University, Changsha, China; 3 Department of Psychiatry, Jiangxi Provincial People’s Hospital, The First Affiliated Hospital of Nanchang Medical College, Nanchang, China; 4 Affiliated Wuhan Mental Health Center, Tongji Medical College, Huazhong University of Science and Technology, Wuhan, China; 5 Xiangya Nursing School of Central South University, Changsha, China; 6 School of Mental Health, Wenzhou Medical University, Wenzhou, China; 7 The Ninth Hospital of Changsha, Changsha, China; 8 Department of Psychiatry, Shandong Provincial Hospital, Shandong University, Jinan, China; 9 RIKEN Center for Advanced Intelligence Project, Tokyo, Japan; 10 Research Center for Advanced Science and Technology (RCAST), University of Tokyo, Tokyo, Japan; 11 Hunan Provincial Brain Hospital (The Second People’s Hospital of Hunan Province), Changsha, China; 12 Zigong Mental Health Center, Zigong, China

**Keywords:** Treatment-resistant schizophrenia, Interleukin-6, Cytokines, Inflammation, Sex

## Abstract

**Background:**

Low-grade inflammation and altered inflammatory markers have been observed in treatment-resistant schizophrenia (TRS). Interleukin-6 (IL-6) is one of the pro-inflammatory cytokines linked with TRS and receives increasing attention. Previous studies showed that patients with TRS might have higher IL-6 levels compared with healthy individuals and treatment-responsive patients. Besides, emerging evidence has suggested that there are sex differences in the associations between IL-6 levels and various illnesses, including chronic hepatitis C, metabolic syndrome, etc.; however, there is limited study on TRS. In this present study, we aimed to compare the serum IL-6 levels of TRS and partially responsive schizophrenia (PRS) and explore potential sex differences in the association of TRS and IL-6 levels.

**Methods:**

The study population consisted of a total of 90 patients with schizophrenia: 64 TRS patients (45.3% males and 54.7% females) and 26 PRS patients (46.2% males and 53.8% females). We measured serum IL-6 levels using enzyme-linked immunosorbent assay (ELISA) and analyzed them separately by gender, controlling for confounders (age, education, medication, body mass index, and PANSS scores) rigorously.

**Result:**

The results showed that patients with TRS had higher serum IL-6 levels than patients with PRS (p = 0.002). In females, IL-6 levels increased significantly in the TRS group compared with the PRS group (p = 0.005). And a positive correlation tendency was observed between IL-6 levels and PANSS general sub-scores (r = 0.31, p = 0.039), although this correlation was not significant after correcting for multiple comparisons. Whereas, there were no differences in IL-6 levels between the TRS and PRS (p = 0.124) in males.

**Conclusion:**

Our findings provided evidence supporting the hypothesis that the inflammatory response system (IRS) may play a role in the pathogenesis of TRS in a sex-dependent manner. In addition, sex differences in the immune dysfunction of individuals with schizophrenia cannot be neglected, and inflammation in male and female TRS should be discussed separately.

## Background

Approximately a third of patients with schizophrenia have limited response to two or more kinds of first-line antipsychotics except for clozapine, which is defined as treatment-resistant schizophrenia (TRS), a specific phenotype of schizophrenia [1–3]. The pathophysiological mechanisms of TRS remain unknown. For decades, numerous pieces of evidence have suggested that both the inflammatory response system (IRS) and compensatory immune-regulatory system (CIRS) play roles in the pathophysiological processes underlying TRS [4, 5]. Several elevated immune biomarkers, including IL-6 (interleukin-6), sIL-1RA (soluble IL-1 receptor antagonist), IL-2 (interleukin-2), IL-10 (interleukin-10), sTNF-R1 (soluble tumor necrosis factor receptor 1), sTNF-R2 (soluble tumor necrosis factor receptor 2), IL-8 (interleukin-8), CCL-3 (chemokine ligand-3), and CCL-2 (chemokine ligand-2), have been found in TRS successively [4, 6–9].

IL-6 is a pro-inflammatory cytokine mainly produced by mononuclear macrophages and microglia in the circulating immune system and the central nervous system, respectively [10], and can affect neuronal, glial, and immune responses for its involvement in regulating brain neurodevelopment and changing synaptic plasticity [11]. IL-6 has been regarded as one of the state cytokine markers of schizophrenia, which can be increased in first-episode psychosis (FEP) and be regulated by antipsychotic treatment [5, 6, 12]. In vitro, the second-generation atypical antipsychotics olanzapine and aripiprazole showed an effect on the reduction in IL-6 concentrations of primary human peripheral blood mononuclear cells (PBMC) from healthy individuals [13]. In addition, it has been noted that elevated IL-6 levels in schizophrenia may contribute to the occurrence of metabolic syndrome [14–16]. However, metabolic syndrome is one of the most common side effects of second-generation atypical antipsychotics. Besides, it has been observed that clozapine increases IL-6 expression in murine pancreatic tissue [16]. So, the effect of antipsychotics on IL-6 levels seems to be controversial, which also suggests that different types of antipsychotics may have different impacts on IL-6. However, so far, there has been no study on the comparisons between the effects of different antipsychotic types on IL-6 levels. IL-6 has been one of the cytokines linked with TRS [4]. In 1998, Lin et al. found that serum IL-6 levels in TRS were significantly higher than in healthy controls [4]. This finding was also supported by subsequent studies [8, 17–21]. While the differences in IL-6 levels between TRS and treatment-responsive schizophrenia were not significant in some of these studies [4, 8]. Nevertheless, there were increasing trends in the IL-6 levels in the patients with TRS compared to the patients with treatment-responsive schizophrenia in these studies. The relatively small sample sizes might contribute to their nonsignificant results. Besides, as in early studies, the medication of patients in those studies might be very different from the medication of patients in subsequent studies.

The impact of gender on circulating IL-6 levels is worthy of note. Even in healthy samples, previous studies on comparisons of IL-6 levels in males and females have shown mixed results, which may be caused by the different samples with different ages, races, etc. [22, 23]. IL-6 in males and females can play different roles in the pathophysiological process of some diseases. A retrospective cohort study showed that elevated serum IL-6 levels were associated with higher incidence rates of hepatocellular carcinoma in female patients with chronic hepatitis C but not in male patients [24]. In ankylosing spondylitis patients, elevated IL-6 levels in patients with syndesmophytes could be observed only in females [25]. Whereas in sleep apnea patients, males rather than females had an elevated IL-6 level compared with healthy control [26]. Emerging studies have shown that sex differences in IL-6 may also occur in patients with schizophrenia. Significantly higher IL-6 levels in patients with deficit schizophrenia were observed in females, but not in males [27]. On the contrary, elevated IL-6 levels could only be observed in male patients with metabolic syndrome and were negatively correlated with their high-density lipoprotein levels [15]. Besides, IL-6 levels were only associated with obesity in female patients treated with clozapine [14]. It has become increasingly clear that gonadal hormones can have an effect on immune functioning [28]. Estrogen-mediated inhibition of IL-6 production [29] and proinflammatory effects of testosterone [30] have been observed, however, they cannot explain why, in some illnesses, IL-6 levels in females are higher than in males.

To our knowledge, most studies on IL-6 levels in TRS just compared patients with TRS with healthy control [16–18, 21, 31]. However, as mentioned before, IL-6 is considered a state cytokine marker of schizophrenia, and increased IL-6 levels have been found in various phenotypes of schizophrenia (e.g., FEP) [5]. So, it is necessary to compare IL-6 levels among the phenotypes, which may provide meaningful information on the identification of TRS. Furthermore, although some previous studies have found that TRS patients had significantly higher IL-6 levels than patients with treatment-responsive schizophrenia [19, 20], possible sex differences have not been expressly discussed yet. Hence, in this study, we aimed to compare the serum IL-6 levels between TRS patients and patients with treatment-responsive schizophrenia and explore potential sex differences in the association of TRS and IL-6 levels.

## Methods

### Participants

The study sample consisted of 90 right-handed inpatients, aged 18 to 49, who met the DSM-V-TR criteria for schizophrenia. Participants were enrolled at the Second Xiangya Hospital of Central South University, the Hunan Provincial Brain Hospital, and the Ninth Hospital of Changsha from 2020 to 2022. Exclusion criteria included substance abuse, a history of head trauma resulting in loss of consciousness, and major medical or neurological illness. We reviewed the medical records of all patients and classified TRS patients versus PRS patients based on their previous responses to antipsychotics. Patients were diagnosed with TRS based on the consensus guidelines published by the Treatment Response and Resistance to Psychosis (TRRIP) Working Group in 2017, in which TRS was defined as schizophrenia that had a poor response (Positive and Negative Syndrome Scale > 60, Global Assessment of Functioning 60) to two different adequate doses (dosage equivalence of chlorpromazine 600 mg/day) and courses (6 weeks) of non-clozapine antipsychotics [32]. Besides, among female patients, no one was menstruating or using contraceptives. This study was approved by the Second Xiangya Hospital Ethics Committee (No. S006, 2018), and all participants fully understood the research procedures and provided written informed consent.

### Clinical evaluation

Clinical evaluation was performed independently by two experienced senior psychiatrists. The severity of illness was assessed by the Positive and Negative Syndrome Scale (PANSS) and Global Assessment of Functioning Scale (GAF).

### Measurement of serum IL-6 levels

On the day of enrollment or the next morning, fasting venous blood was collected and placed in a non-anticoagulant vacuum tube. The blood samples were rested and centrifuged (3000 rpm for 10 min), and the serum was extracted and stored in an ultra-low temperature refrigerator (-80 °C). Serum IL-6 was measured by the quantitative enzyme-linked immunosorbent assay (ELISA) method using commercially available kits (R&D Systems Europe Ltd., Abingdon, UK). Each sample was tested twice, and the final results were averaged from the two assay values.

### Statistical analysis

All statistical analyses were performed using SPSS (version 25 IBM Inc. New York, USA). Means ± standard deviations (X ± SD) were used for measurement data. A two-tailed p-value < 0.05 indicated statistical significance. Two-tailed independent-sample t-tests were used to compare the demographic data, including age, PANSS score, GAF score, duration of disease, medication dose, and BMI, between males in the TRS and the PRS group and females in the TRS and the PRS group (within gender). Shapiro–Wilk’s tests were used as normality tests, and the data of IL-6 levels did not fall into a normal distribution; therefore, Spearman correlations were used to explore the relationship between IL-6 levels and years of education, duration of illness, dose of medication, or BMI of the two male subgroups, the two female subgroups, and the two overall groups. We first investigated IL-6 levels in the two overall groups, then we compared IL-6 levels in females with males in the same group and females or males in the different groups. The generalized linear model (GLM) was used to compare the differences in IL-6 levels between the two groups, the two male subgroups, the two female subgroups (within gender), between males and females (between gender), or between patients in the TRS group with and without clozapine treatment (within gender). Due to the differences in demographic and clinical characteristics and their potential associations with IL-6, we included age, medication, education, duration, BMI, and PANSS total scores as covariates. Partial correlational analyses were used to explore the relationship between IL-6 levels and PANSS scores or clozapine dose. FDR correction was performed for multiple comparisons.

## Results

### Demographic and clinical characteristics

Sixty-four patients with TRS and 26 patients with PRS were enrolled in this study. The demographic and clinical characteristics of the male and female participants are summarized in Table 1. Within gender, there were no significant differences in age and medication among the two groups. Patients in the PRS group had a shorter duration of illness than in the TRS group (p = 0.023 for males, p = 0.003 for females). In male patients, the PRS group had a longer education year compared with the TRS group (p = 0.001), but this difference was not observed in female patients (p = 0.196). A higher BMI was observed in female TRS patients (p = 0.003) but not in males (p = 0.834). In terms of clinical characteristics, male patients and female patients were similar. Both in males and females, TRS patients had higher PANSS sub-scale scores and total scores (all ps < 0.05) and lower GAF scores (p = 0.001 for males and p = 0.001 for females).

**Table 1:** Demographic and clinical characteristics of patients with TRS and patients with PRS, stratified by gender

	Male			Female		
	TRS (n = 29)	PRS (n = 12)	*p* value	TRS (n = 35)	PRS (n = 14)	*p value*
Age (years)	26.14 ± 6.31	22.50 ± 4.76	0.054	37.80 ± 9.02	34.00 ± 7.83	0.174
Education(years)	10.14 ± 2.56	13.08 ± 1.68	0.001**	10.94 ± 2.88	12.11 ± 2.62	0.196
Duration of illness (years)	7.69 ± 3.98	4.58 ± 3.42	0.023*	15.91 ± 9.93	8.29 ± 6.43	0.003**
Medication (CPZE mg/day)	667.76 ± 234.62	565.42 ± 271.26	0.232	473.71 ± 228.06	425.36 ± 190.97	0.456
BMI (kg/m^2^)	24.88 ± 6.07	24.42 ± 6.75	0.834	25.88 ± 5.21	22.34 ± 2.53	0.003**
PANSS Positive	19.07 ± 6.58	14.00 ± 4.05	0.018*	20.94 ± 7.71	12.79 ± 6.19	0.001**
PANSS Negative	21.45 ± 7.82	14.08 ± 4.83	0.004**	19.20 ± 9.37	9.43 ± 3.46	< 0.001***
PANSS General	34.45 ± 7.66	22.75 ± 5.19	< 0.001***	36.83 ± 7.37	21.57 ± 4.62	< 0.001***
PANSS Total	75.72 ± 14.90	50.83 ± 8.89	< 0.001***	76.97 ± 16.23	43.64 ± 8.32	< 0.001***
GAF	50.59 ± 13.29	66.67 ± 4.58	0.001**	48.97 ± 17.44	68.57 ± 9.25	< 0.001***
IL-6 (pg/ml)	2.49 ± 1.53	1.14 ± 1.86	0.124	2.61 ± 1.78	0.75 ± 0.76	0.005**

*Note*: Values are provided as mean ± SD unless otherwise stated. BMI, body mass index; PANSS, Positive and Negative Syndrome Scale; GAF, Global Assessment of Functioning scale; CPZE, chlorpromazine equivalent dose; IL-6, interleukin 6

We examined the association of IL-6 levels with demographic indicators including age, medication, duration of illness, and BMI in men and women. There were no significant correlations in the two female groups (all p > 0.082). For males, a positive correlation between IL-6 levels and BMI was observed in the TRS group (r = 0.462, p = 0.012), but not in the PRS group (r = 0.049, p = 0.880). No correlations between IL-6 and other indicators were found in either the TRS group or the PRS group (all p > 0.199). Because of these basal sex differences and potential effects, we included these indicators as covariates in the following analyses.

### Group comparisons of serum IL-6 levels

**Fig. 1:**
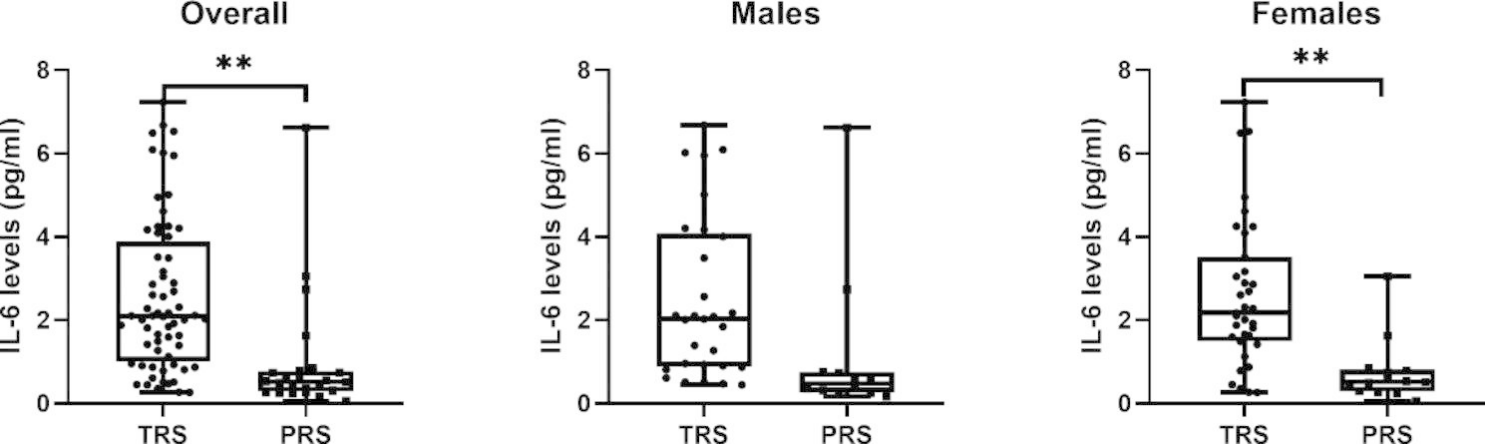
Serum IL-6 levels (mean and SD) of TRS and PRS. Abbreviations: TRS, patients with treatment-resistant schizophrenia; PRS, partially responsive schizophrenia; IL-6, interleukin-6. **p < 0.01

Serum IL-6 levels of the two groups stratified by gender are presented in Table 1; Fig. 1. Overall, IL-6 levels were significantly higher in the TRS group (2.56 ± 1.85 pg/ml) than in the PRS group (0.93 ± 1.36 pg/ml) (p = 0.002), even after including gender as a covariate (p = 0.02). Females in the TRS group had significantly higher IL-6 levels than in the PRS group (p = 0.005). However, no differences in IL-6 levels between the TRS and the PRS groups were observed in males (p = 0.124). Regarding gender, we also controlled for age, medication, education, duration, BMI, and PANSS total scores, and neither the TRS group (p = 0.838) nor the PRS group (p = 0.053) showed differences in IL-6 levels between males and females.

### Correlation between symptom severity and serum IL-6 levels

**Fig. 2:**
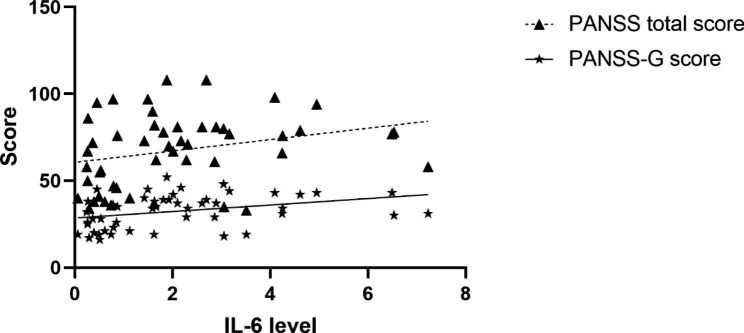
Correlations between serum IL-6 levels with PANSS-G scores and PANSS total scores in female patients. Abbreviations: IL-6, interleukin-6; PANSS-G, General Psychopathology scale

In female patients, serum IL-6 levels were significantly associated with PANSS general sub-scores (r = 0.31, p = 0.039) (Fig. 2). Nevertheless, after applying FDR correction, the above correlation failed to pass the correction (FDR-corrected q = 0.156). Besides, a trend toward a correlation between PANSS total scores and IL-6 levels (r = 0.27, p = 0.070) was also observed (Fig. 2). However, when we analyzed females in the two subgroups separately, no correlations were found. Whereas for male patients, there was no significant correlation between IL-6 levels and PANSS sub-scores or total scores in the two subgroups, nor in all male patients.

### The effect of clozapine on serum IL-6 levels in TRS

All patients with TRS have been recommended to receive clozapine treatment; however, some of them have refused. Therefore, the TRS group could actually be divided into two sub-groups according to the administration of clozapine. First, we compared IL-6 levels in TRS patients with (17 males and 17 females) or without (12 males and 18 females) clozapine treatment, and no significant differences were found either in males (p = 0.382) or in females (p = 0.482). In addition, correlation analyses were conducted in clozapine-treated patients. The mean daily clozapine dose for males and females was 277.94 ± 79.00 mg/day and 251.47 ± 85.45 mg/day, respectively. No significant correlations between the daily clozapine dose and IL-6 levels were found in males (p = 0.348) or females (p = 0.369).

## Discussion

In this study, we investigated serum IL-6 levels in patients with TRS and with PRS and analyzed them separately by gender. After controlling for confounders rigorously, we found that, in females, IL-6 levels were significantly higher in patients with TRS compared with patients with PRS, and a positive correlation tendency was observed between IL-6 levels and symptom severity. On the contrary, there were no differences in IL-6 levels between the two male groups. Consistent with our expectation, sex differences in the association of TRS and serum interleukin-6 levels were observed.

Our findings in female patients are supported by previous studies [4, 8, 19, 20], which indicated elevated serum IL-6 levels were associated with TRS. As an element of IRS activation, IL-6 has been linked with many diseases, including obesity and schizophrenia [33]. In previous studies, elevated IL-6 levels were usually associated with more severe symptoms and cognitive deficits in schizophrenic patients [18, 34, 35]. However, what causes the increase in IL-6 levels and what role IL-6 plays in the pathogenesis of schizophrenia remain unclear. It has been suggested that risk factors for schizophrenia, including pre and perinatal infection, childhood adversity, and exposure to infectious agents, might contribute to inflammatory abnormalities then increased IL-6 levels [10, 34, 36, 37]. A Mendelian randomization study showed that genetically predicted IL-6 levels were associated with brain structure in some regions highly associated with schizophrenia [38]. It is widely accepted that second-generation atypical antipsychotics may have anti-inflammatory effects on the reduction of the IL-6 levels in schizophrenic patients, and be accompanied by an improvement in their symptoms [10, 13, 39]. Therefore, we assumed that persistently elevated IL-6 levels might cause refractory symptoms and that the anti-inflammatory effects of antipsychotics might fail in female patients with TRS. However, as mentioned earlier, the previous studies on the effects of antipsychotics on IL-6 levels showed inconsistent results, which might be due to the different antipsychotics and subjects in the studies. It has been suggested that clozapine can have an effect on serum IL-6 levels in patients with schizophrenia [14, 16, 40], so we also explored the relationship between clozapine and serum IL-6 levels in the TRS group. Nevertheless, our results were based on a relatively small sample size, which needs to be verified in further studies with large samples. In addition, the patients in our study were not on clozapine monotherapy, and the other antipsychotics could have various impacts on the serum IL-6 levels, which might also overshadow the effects of clozapine. Hence, further studies are required to explore the effects of different antipsychotics on pro-inflammatory cytokine levels in patients with various subtypes of schizophrenia, and patients on antipsychotic monotherapy should be considered as the subjects.

It is well-established that sex differences in inflammatory markers are widespread among various populations, including healthy individuals [26, 41]. In previous studies on patients with schizophrenia, sex differences in IL-6 levels have been observed in patients with deficit schizophrenia and metabolic syndrom [15, 27]. Although most previous studies on IL-6 levels in TRS controlled for sex as a confounder in their statistical analyses, there were few studies specifically discussing the possible sex differences. In fact, our results were similar to the study on deficit schizophrenia, which also showed higher IL-6 levels in female deficit schizophrenia patients compared with nondeficit schizophrenia patients, and no differences between male patients [27]. There were some overlaps with the subjects of the two studies, TRS and deficit schizophrenia. In addition, despite the fact that we did not measure more indicators related to metabolism, we did observe a correlation between IL-6 levels and BMI was observed in male TRS patients, but not in females. However, the biological mechanisms underlying the sex differences in the association of IL-6 levels with more severe schizophrenia or metabolic aberrations remain unclear [27]. Different IL-6 levels in males and females have been explained as the outcomes of different sex hormone levels [42, 43]. Numerous studies have revealed that estrogen might be a protective factor for immune status because of its anti-inflammatory effect [44]. Meanwhile, sex-steroid-nervous system interactions may be related to schizophrenia. It has been suggested that estrogens can modulate the activity of multiple neurotransmitters associated with schizophrenia, including dopamine, serotonin, and glutamate. Female patients with schizophrenia showed reduced circulating levels of estradiol and adjunctive oestrogen therapy have proved to be effective on female patients with TRS in clinical trials [45, 46]. Hence, we speculated that sex hormones and IL-6 might play different roles in the pathophysiological processes of schizophrenia in males and females. Women with TRS might have reduced estradiol levels, which contribute to their elevated IL-6 levels. However, our study did not measure sex hormone levels, so we were unable to explore the relationship between sex hormones and IL-6 further. The existing research about the sex differences in the association of schizophrenia with elevated IL-6 levels or other immune responses is rare, and further study is needed.

There are several limitations to this study. First, limited by the specificity of the study subjects, the sample size was small. And it was also difficult to recruit TRS and PRS groups of subjects with matched illness duration, education, medication, and BMI. Furthermore, the demographic and clinical characteristics of male and female patients were not completely consistent (i.e., the severity of psychiatric symptoms). Hence, we chose stratification analysis to explore the sex differences and controlled for confounders including PANSS scores rigorously. However, the sample size was still small for a stratification analysis, which might be a reason for the insignificant results (i.e., the correlation between IL-6 levels and PANSS in females), so our findings should be validated in future studies with large sample sizes and matched groups. In addition, the study subjects have a relatively long duration of illness and are old, which might impact IL-6 levels. Therefore, in the data analyses, we controlled for these confounders rigorously. However, considering the broad effects of IL-6, there were still many confounders difficult to exclude in a cross-sectional study. Hence, the inclusion of healthy controls and first-episode schizophrenia patients and longitudinal studies should be considered in future studies.

## Conclusions

In summary, we found that increased IL-6 levels were associated with female TRS in females but not in males. Our findings may provide evidence to support the involvement of inflammation with TRS in female patients and the immune-inflammatory effect of IL-6 on TRS may be in a sex-dependent manner. Antagonists of IL-6 and anti-inflammatory drugs can be prominent targets for the treatment of female TRS in the future. It is also emphasized that sex differences in the immune dysfunction of individuals with schizophrenia cannot be neglected, and inflammation in male and female TRS should be discussed separately.

## Data Availability

The datasets used and analysed during the current study available from the corresponding author on reasonable request.
